# Accelerated Bioconversion of Chemically Solubilized Lignite Solution to Methane by Methanogenic Consortium: Experimental Results and Their Application to the Subsurface Cultivation and Gasification Method

**DOI:** 10.3390/microorganisms10101984

**Published:** 2022-10-07

**Authors:** Akio Ueno, Satoshi Tamazawa, Shuji Tamamura, Takuma Murakami, Tamotsu Kiyama, Hidenori Inomata, Noritaka Aramaki, Kunihiko Yoshida, Shinji Yamaguchi, Hideo Aoyama, Takeshi Naganuma, Toshifumi Igarashi

**Affiliations:** 1 Horonobe Research Institute for the Subsurface Environment (H-RISE), Northern Advancement Centre for Science and Technology (NOASTEC), Horonobe 098-3221, Japan; 2 National Institute of Technology (KOSEN), Kagawa College, Takamatsu 761-8058, Japan; 3 Mitsubishi UBE Cement Corporation (MUCC), Tokyo 100-8521, Japan; 4 Graduate School of Integrated Sciences for Life, Hiroshima University, Hiroshima 739-8527, Japan; 5 Faculty of Engineering, Hokkaido University, Hokkaido 060-8588, Japan; 6 National Institute of Technology (KOSEN), Asahikawa College, Hokkaido 079-8501, Japan

**Keywords:** subsurface cultivation and gasification method, lignite, conductive materials, hydrogen peroxide, biogenic methane, methanogens, *Methanosarcina*, bioaugmentation

## Abstract

Lignite is an obsolete and less commercially circulated natural resource due to its low calorific value worldwide. The effective conversion of lignite into methane is important considering the global energy crunch. This study reported the effective bioconversion of organic matter released from chemically solubilized lignite to methane using two methanogenic consortia types: mixed methanogenic enrichment culture (mMEC) and SAL25-2. We demonstrated in a microcosm study that the start of methane generation was observed within seven days. Furthermore, the methane yield increased as the total organic carbon concentration of the chemically solubilized lignite solution increased. Surprisingly, methane production using mMEC was drastically enhanced by approximately 50–fold when pulverized lignite was added as conductive material (CM) to the microcosms. To the best of our knowledge, this is the highest number of times methane production increased relative to the control. Our results demonstrated that bioaugmentation using a methanogenic consortium and adding pulverized lignite as CM could facilitate the bioconversion of chemically solubilized lignite solution to methane and lead to effective utilization of subterranean lignite, regarded as a neglected natural resource, without any further excavation processes.

## 1. Introduction

Coal is one of the most utilized fossil fuels and a worldwide resource and has been utilized since the Industrial Revolution [1]. Approximately 71.4% of global fossil fuel reserves are coal [2]. Coal is generally not regarded as a favorable microbial substrate because of its complex and recalcitrant molecular structure, with a limited fraction of biodegradable moieties (such as phenolics, carboxylic acids, and alkanes) [3]. Lignite, which is often referred to as brown coal, is classified as low-rank coal that is formed from the original phytomass by peatification followed by coalification [4,5]. The main original materials of low-rank coals, including lignite, are reported to have a lignin-like polymer structure and lignocellulosic biomass [3]. Consequently, the effects of lignolytic and hydrolytic microorganisms on coal degradation have been extensively studied [6].

Lignite is an intermediate between peat and bituminous coal and is less utilized and commercially circulated worldwide than its higher-ranked counterparts [7,8]. Owing to its low calorific value and typically high moisture content, lignite is inefficient for transportation and is not traded extensively in the world market compared to higher coal grades, such as bituminous and sub-bituminous coals. Although lignite has many disadvantages in industrial use, it undergoes biological degradation more easily than higher-ranked coals. This unique feature has triggered interest in using lignite as a nontraditional fuel [9]. Since the discovery of biogenic gas generation in coal bed reservoirs and the development of geochemical methods to distinguish biogenic and thermogenic gases, coal beds harboring microbial gas (biogenic coalbed methane (CBM)) have been found worldwide [10,11]. Biogenic CBM production generally occurs at shallow depths at temperatures less than 100 °C, as reported in the San Juan [12], Powder River [13,14,15], Illinois [16,17], and Fort Yukon basins [14] in the United States, the Surat Basin [18] in Australia, the Ruhr Basin [19] in Germany, the Ordos, Qinshui, and Jingmen-Dangyang basins [20,21,22] in China, and the Yubari coal field [23] in Japan. Additionally, it has been shown that biogenic CBM occurred in relatively recent generations (under 50,000 years) by hydrological and isotopic studies in the Powder River [24] and Illinois [25] basins in the United States, and the Surat Basin in Australia [26]. These findings suggest that biogenic methane production is an ongoing process worldwide.

Coal methanogenesis requires a complex community of bacterial and archaeal species to cooperatively degrade the complex coal matrix via a sequence of fermentation and syntrophic interactions [12]. Within coal beds, organic compound-degrading bacteria likely provide methanogens with the necessary substrates, including acetate, CO_2_, and H_2_ to produce methane [11]. Although much of the process remains unsolved, it has been hypothesized that soluble organic molecules (long-chain fatty acids, alkanes, and low molecular weight aromatics) are first released from coal [27], followed by biodegradation into substrates (acetate, CO_2_ and H_2_, methanol, and formate) that can be utilized by methanogens, with CH_4_ and CO_2_ being produced by methanogens [28]. Methanogens can utilize a few substrates for methanogenesis, which include acetate, CO_2_, H_2_, some C1 compounds (formate, methanol, methylamines, and methylthiols) [29,30,31,32,33,34,35], and methoxylated compounds [36]. This means that the activity of methanogens is limited by the availability of substrates that can be utilized by methanogens, and the generation of those substrates from coal appears as the rate-limiting step in biomethane production.

Hydrogen peroxide-induced coal solubilization has been shown to produce dissolved organic matter [37,38,39,40,41,42]. Successful production of organic acids from coal has been made [9,18,43,44,45]. However, a handful of research groups have attempted microbial augmentation at the field scale [10]. In previous studies, we proposed a methane generation system utilizing the terrestrial subsurface environment, the Subsurface Cultivation, and Gasification (SCG) method [46,47].

This article presents the results of biomethane production from chemically solubilized lignite solution by bioaugmentation, that is, adding a methanogenic consortium and pulverized lignite as conductive materials (CM). We discuss the possibility of facilitating biomethane production after the solubilization of lignite with H_2_O_2_.

## 2. Materials and Methods

### 2.1. Sample Collection and Preparation of Chemically Solubilized Lignite Solution

Lignite samples were collected from Tempoku Coalfield, which is located in the northernmost part of Hokkaido, Japan. The coalfield stretches approximately 60 km from north to south and approximately 20 km from east to west [48]. This coalfield harbors several coal seams called Soya coal-bearing formations, comprising lignite, coaly shale, and tuff [49], forming one of the largest lignite deposits in Japan (recoverable resources are 10^9^ tons) [45]. Lignite samples were collected from a riverbed outcrop at the Teshio Experimental Forest of Hokkaido University. The sampling site, total organic carbon (TOC) concentration, volatile matter content, and calorific value of the sampled lignite have been described previously [50]. This lignite sample was used to prepare a chemically solubilized lignite solution, as described below, and pulverized lignite was used as CM. The pulverized lignite was prepared by sieving the crushed lignite (<1 mm), and was stored at 4 °C until use.

Chemically solubilized lignite solutions were prepared according to a previous report [45]. The characteristics of the original solutions used in this study are listed in Table 1. The dissolved TOC concentration was 2800 mg/L TOC, having a pH value was 2.4. The concentration of residual H_2_O_2_ was <10^−4^%. This solution was filter-sterilized using a 0.22 µm-pore filter before use and was then added to culture media in microcosms with three different concentrations of 10, 100, and 1000 mg/L TOC, as described in Section 2.3.

### 2.2. Construction of Methanogenic Enrichment Cultures

We routinely maintained five types of methanogenic enrichment cultures (MECs) constructed from groundwater samples in our laboratory. These MECs were designated as numbers 35, 36, 37, 45, and 46, and their origins are reported below. The No. 35 and 36 MECs originated from a groundwater sample at 288.7–303.0 and 362.4–385.7 m below ground level (mbgl) in Horonobe deep boreholes (HDB)-6 drilled by the Japan Atomic Energy Agency (JAEA), respectively [51]. MEC No. 37 originated from a groundwater sample at 606.0–644.1 mbgl in HDB-11 [51]. MEC No. 45 originated from a groundwater sample collected from the NS15 borehole of one of three separators in a gas-petroleum reservoir in Higashi Niigata, Japan [52]. MEC No. 46 originated from a groundwater sample collected from one production well PW-1 in the Yubari enhanced CBM recovery site located to the south of Ishikari coal field, Hokkaido, Japan [23]. Each MEC was separately transferred to new media at intervals of approximately two months (Figure S1). Cultivation of the “Old enrichment” started on 27 June 2017: “Enrichment #1” on March 6, 2018, “Enrichment #2” on 27 April 2018, and “Enrichment #3” on 28 June 2018 (Figure S1). All MECs were incubated at 30 °C in the dark.

### 2.3. Microcosm Experiments

Microcosms were set up using the anaerobic tubes with butyl rubber stoppers (size 18 mm internal diameter × 180 mm length, Sanshin Industrial Co., Ltd., Yokohama, Japan) containing 10 mL of anaerobic medium as described below to examine the potential for methane production from the organic matter released from chemically solubilized lignite pre-treated with H_2_O_2_. The anaerobic medium contained the following (in g/L): NH_4_Cl (0.5), MgCl_2_-6H_2_O (0.5), CaCl_2_-2H_2_O (0.14), K_2_HPO_4_ (0.14), KCl (0.1), NaCl (0.6), Fe (NH_4_)_2_ (SO_4_)_2_-6H_2_O (0.002), and NaHCO_3_ (2.5). Moreover, the medium contained 0.001% (wt./vol.) resazurin, 0.05% Na_2_S, and 1 mL/L of trace mineral solution SL-10. The trace mineral solution SL-10 contained the following (in mg/L): FeCl_2_-4H_2_O (1,500), ZnCl_2_ (70), MnCl_2_-4H_2_O (100), H_3_BO_3_ (6.0), CoCl_2_-6H_2_O (190), CuCl_2_-2H_2_O (2.0), NiCl_2_-6H_2_O (24), Na_2_MoO_4_-2H_2_O (36), and 10 mL of 25% (7.7 M) HCl. Finally, the chemically solubilized lignite solution was added to final concentrations of 10, 100, or 1000 mg/L TOC. When the solid phase was tested as a conductive material (CM), 1 g of pulverized lignite was added to the anaerobic culture medium. The pH of the medium was adjusted to 7.0–7.5 with 5.0 N NaOH because the pH of chemically solubilized lignite solution was 2.4, and adding this solution easily lowered the medium pH. The pH of the medium was checked with a pH meter (Horiba, Japan). The headspace of the anaerobic tubes was filled with anoxic N_2_:CO_2_ (80:20 vol./vol.) gas using a gas exchanger model GR-8 (Sanshin Industrial Co., Ltd., Yokohama, Japan). The headspace volume was 19 mL. As a microbial inoculum, we inoculated the mixed MECs comprising the five types of MECs described above (mMEC) (Figure S2). An equal volume of culture medium was taken from each enrichment culture, mixed, centrifuged, and then rinsed with a fresh culture medium for the microcosm experiments described below with no organic matter to prevent the carryover of organic matter to the microcosms. Groundwater in borehole “Br. No. 2” was obtained using a sampling device, as described previously [53]. When the microbial consortium originated from the groundwater in borehole “Br. No. 2”, which was named “SAL25-2,” an aliquot of Br. No. 2 was added to the culture medium (groundwater: culture medium = 1:9 vol./vol.), supplemented with chemically solubilized lignite solutions with different concentrations of 10, 100, or 1000 mg/L TOC. The pH of the medium was adjusted to 7.0 using 5.0 M NaOH. The headspace of the anaerobic tubes was filled with anoxic N_2_:CO_2_ (80:20 vol./vol.) gas. All microcosm experiments were conducted at 30 °C in the dark. The microcosm numbers were assigned to be No. 1-11 when mMEC was used, whereas they were assigned to be No. 12-22 when SAL25-2 was used.

### 2.4. Analytical Procedures

The methane concentration in the headspace of the microcosms was analyzed by gas chromatography (GC) using a gas chromatograph model GC-14B with a flame ionization detector (FID) (Shimadzu, Kyoto, Japan) equipped with a 30-m 0.25-mm-ID 0.25-m-film-thickness Rtx-5 capillary column (Restec Col., Ltd., Bellefonte, PA, USA). Helium was used as the carrier gas. The analytical conditions have been previously described [53]. For methane analysis, 100 μL of the headspace of the anaerobic tubes was collected with a 500 μL gas-tight sample lock syringe (model 1750SL, Hamilton, NY, USA) through butyl rubber stoppers and injected into the gas chromatograph. Methane production [µmol] was calculated by converting peak areas of methane detected by GC analysis using an external standard curve as described previously [53]. Total carbon content in CH_4_ production [µg C] was calculated according to the following equation:

Total carbon content in CH_4_ production [µg C] = CH_4_ production [µmol] × 16.04 (molecular weight of CH_4_) × 12/16.04 (ratio of carbon content in CH_4_).

The conversion rate of TOC to methane was calculated according to the following equation:

Conversion rate [%] = Total carbon content in CH_4_ production [µg C] / TOC in 10 mL culture [µg C] × 100.

### 2.5. DNA Extraction and Next-Generation Sequencing

DNA was extracted from the culture media using a PowerSoil DNA Isolation Kit (MO BIO Laboratories, Inc. Carlsbad, CA, USA), according to the manufacturer’s protocol. To analyze the taxonomic composition of the groundwater microbial community, the V4 region of the bacterial and archaeal 16S rRNA gene, which corresponds to *Escherichia coli* positions 515–806, was chosen [54,55] and amplified using a two-step protocol according to the instructions of FASMAC, Japan. All procedures were performed following a previous study [53]. All DNA samples were sent to Fasmac Co., Ltd., Atsugi, Japan, using the Illumina platform for Next-Generation Sequence (NGS) service. Raw sequences were denoised and processed using QIIME 2 pipeline ver. 2021.4.0 [56]. In summary, paired-end sequences were joined and denoised using DADA2 [57]. Qualified sequences were clustered into amplicon sequence variants (ASV) [58]. Finally, ASVs were taxonomically classified using the SILVA 138-99 non-redundant ribosomal RNA gene database. For alpha diversity analysis, indices including Chao1, Pielou evenness (*J*’), Shannon–Wiener (*H*’), Simpson diversity (λ), and Faith’s phylogenetic diversity were calculated using QIIME 2 [56]. For beta diversity analysis, principal coordinate analysis (PCoA) was conducted using the R software package. All graphs were visualized using the QIIME2 View interface (https://view.qiime2.org/ (accessed on 15 May 2022)).

### 2.6. Real-Time Quantitative PCR

Real-time qPCR was conducted using a QuantStudio3 Real-Time PCR system (Thermo Fisher Scientific, Waltham, MA, United States). To quantify the *mcrA* gene of methanogens, a specific primer set of MLf (5′-GGTGGTGTMGGATTCACACARTAYGCWACAGC-3′) and MLr (5′-TTCATTGCRTAGTTWGGRTAGTT-3′) was used [59]. To quantify the bacterial 16S rRNA gene, a specific primer set of 341f (5′-CCTACGGGAGGCAGCAG-3′) and 534r (5′-ATTACCGCGGCTGCTGG-3′) was used [60]. Each reaction mixture (20 µL) comprised 10 µL of 2 × PowerUp SYBR Green Master Mix (Thermo Fisher Scientific, Waltham, MA, United States), 0.5 µL of each primer, and 1 µL of DNA template containing approximately 1–10 ng of DNA. A negative control was run using sterilized distilled water as the template instead of a DNA sample. The amplification was initiated by the activation of uracil-DNA glycosylase (UNG) at 50 °C for 2 min and polymerase activation at 95 °C for 10 min, followed by 40 cycles of denaturation at 95 °C for 15 s and annealing and extension at 60 °C for 1 min. Standard curves were created using a 10-fold dilution series of plasmid DNA containing the *mcrA* gene from *Methanosarcina horonobensis* strain HB-1^T^ (accession No. CP009516), or the 16S rRNA gene of *Escherichia coli* strain DSM 18039 (accession No. U00096).

### 2.7. Statistical Analysis

All experiments were conducted in triplicate, and the mean value and standard deviation (Std.) were calculated using Excel software (Microsoft). A *p* < 0.05 was considered statistically significant.

### 2.8. Sequence Accession Numbers

**Figure 1:**
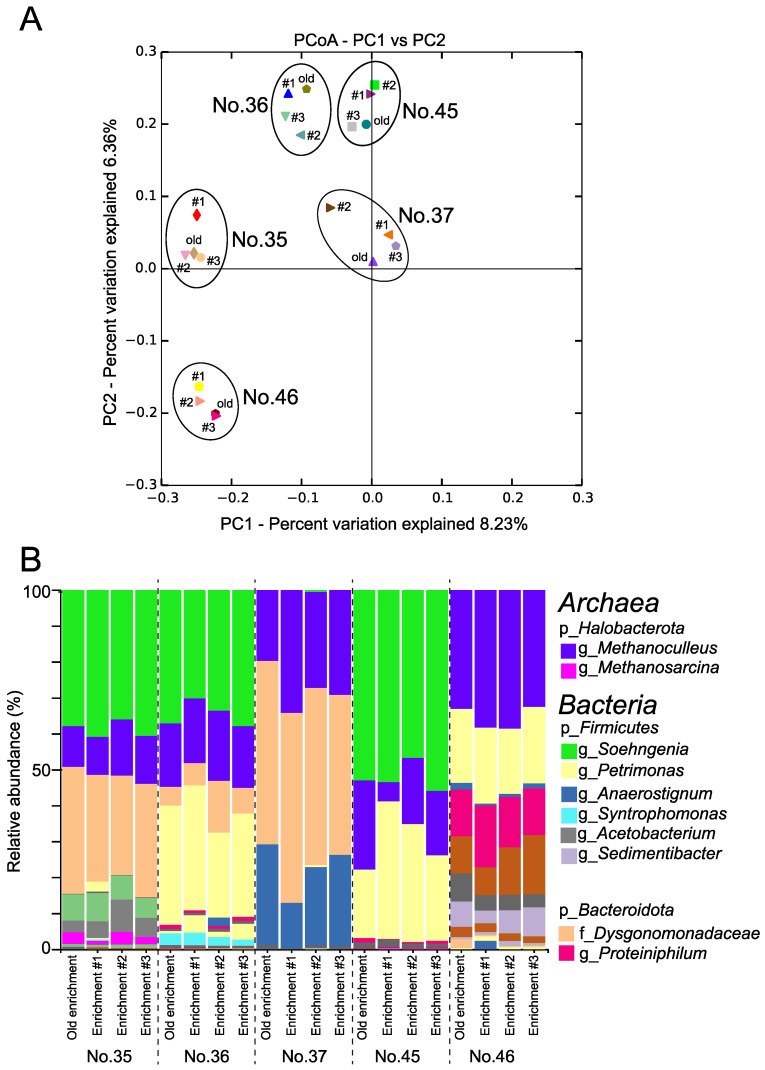
Microbial community structure of the five kinds of methanogenic enrichment cultures (MECs). (**A**) Principal coordinates analysis (PCoA) of the “Old” and the last 3 passages of each enrichment culture (#1, 2, and 3); (**B**) Phylogenetic distribution of each MEC based on the 16S rRNA gene analysis. Major taxonomic groups with a relative abundance of >c.a. 1.0% were indicated. p—Phylum level; f—Family level; g—Genus level.

Raw pyrosequence data were submitted to the DNA Data Bank of Japan (DDBJ). BioProject Accession Number PRJDB12850. The Sequence Read Archive (DRA) accession numbers for Figures 1, 3 and 5 are DRA013976, DRA013977, and DRA013978, respectively.

## 3. Results

### 3.1. Methane Production in Microcosms Supplemented with Chemically Solubilized Lignite Solution Using the Microbial Consortium, mMEC

#### 3.1.1. Characteristics of the MECs

The microbial community structure of the old and the last three passages of the respective MECs, namely No. 35, 36, 37, 45, and 46 MECs, was compared by pyrosequencing. The results are presented in Figure 1 and Supplementary Tables S1 and S5. Principal coordinate analysis (PCoA) showed that each MEC formed a distinctive cluster, thus the microbial community structure of each MEC was maintained in the past year (Figure 1A).

The No. 35, 36, 37, 45, and 46 MECs mainly comprised three phyla: the phylum *Halobacterota* in the domain *Archaea*, and the phyla *Bacteroidota* and *Firmicutes* in the domain *Bacteria* (Figure 1B). The number of ASVs ranged from 3 to 6 in the domain *Archaea* and from 3 to 21 in the domain *Bacteria* (Supplementary Table S1). The Shannon-Wiener (*H*’) and Simpson diversity (λ) indices of No. 37 and 45 MECs were relatively lower than those of No. 35, 36, and 46 MECs (Supplementary Table S1).

The archaeal community analysis revealed that the dominant archaeal sequences belonged to the phylum *Halobacterota* with a relative abundance ranging from 5.4% (minimum, No. 45 Enrichment #1)to 38.6% (maximum, No. 46 Enrichment #2), which consisted of the genera *Methanoculleus* and *Methanosarcina*. The genus *Methanoculleus* was observed in all MECs, with a relative abundance ranging from 5.3% (minimum, No. 45 Enrichment #1) –38.6% (maximum, No. 46 Enrichment #2). In contrast, the genus *Methanosarcina* was detected only in the No. 35 MEC, with a relative abundance ranging from 1.1–3.5% (Supplementary Table S5).

The bacterial community analysis revealed that the relative abundance of the phylum *Bacteroidota* ranged from 20.4–53.0%, whereas that of the phylum *Firmicutes* ranged from 12.9–57.0%. The second dominant bacterial sequence was the genus *Tissierella*, which was detected mainly in the No. 35, 36, and 45 MECs, with a higher relative abundance ranging from 46.3–55.1% in the No. 45 MEC. The genus *Acetobacterium* was detected mainly in No. 35 MECs, with the relative abundances ranging from 3.3–9.0%, and in No. 37 and 45 MECs, with the relative abundances of less than 0.024% (Supplementary Table S5).

#### 3.1.2. Methane Production Using the mMEC

**Figure 2:**
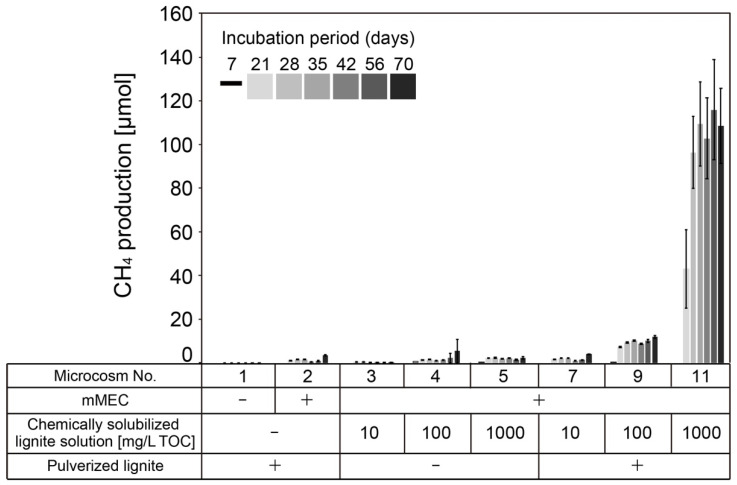
Methane production in the microcosms using mMEC. Values are the mean of three replicates indicating the amount of methane that accumulated in the headspaces of the culture tubes (please refer to Supplementary Table S2). Errors bars (where visible) represent the standard deviation. Microcosm numbers correspond to those in Figures 3 and 6, and Supplementary Tables S2, S3 and S6. The results of microcosms No. 6, 8, and 10 were not shown because mMEC was not added to those microcosms (no methane production was observed). −, not added or absent; +, added or present.

Methane production was observed in the microcosms amended with the chemically solubilized lignite solution (Figure 2 and Supplementary Table S2). As shown in Figure 2, methane production started after 7 days, although the yield was still low (microcosm No. 4 and 5). These results clearly show that the chemically solubilized lignite solution is converted to methane by the mMEC. We examined the methane production in microcosms supplemented with different concentrations of 10, 100, and 1000 mg/L TOC. Methane production was observed in microcosms No. 4 and 5, with a maximum yield of 5.4 ± 5.4 µmol on day 70 in microcosm No. 4. However, the difference in methane production between them was negligible. Little to no methane production was observed in microcosm No. 3. These results showed that chemically solubilized lignite solutions with ≤ 0 mg/L TOC would not produce methane.

Contrary to microcosms No. 3, 4, and 5, higher methane production was observed when pulverized lignite was added to the microcosms (microcosms No. 9 and 11). The microcosms containing only 1 g of pulverized lignite showed little to no methane production (microcosm No. 1), suggesting that microorganisms indigenous to the pulverized lignite added as a solid phase had little to no ability to produce methane. Surprisingly, the addition of pulverized lignite to microcosms No. 9 and 11 enhanced methane production compared to the samples without pulverized lignite in microcosms (No. 4 and 5), although the same amount of chemically solubilized lignite solution was added (Figure 2). The methane production was not enhanced in microcosm No. 7, to which the chemically solubilized lignite solution with 10 mg/L TOC was added. In microcosm No. 9, the maximum methane production was 11.9 ± 0.5 µmol on day 70. The highest methane production was observed in microcosm No. 11, with a maximum of 115.9 ± 22.9 µmol on day 56. This maximum value was approximately 10 times higher than that in microcosm No. 9.

The conversion rate of TOC to methane was calculated and the results are summarized in Supplementary Table S2b,c. The conversion rate of TOC to methane in microcosms No. 3, 4, and 5, where pulverized lignite was not added, ranged from 0–2.8%, whereas that in microcosms No. 7, 9, and 11, where pulverized lignite was added, ranged from 0–13.8% (Supplementary Table S2c).

Microcosm No. 5 was regarded as the control experiment for microcosm No. 11 in terms of the effect of the addition of pulverized lignite as a CM on methane production. The maximum values of methane production in the microcosms No. 5 and 11 were 2.3 ± 0.5 µmol on day 70 and 115.9 ± 22.9 µmol on day 56, respectively. This means that the methane increase was approximately 50–fold when pulverized lignite was added as a CM to the microcosms.

#### 3.1.3. Microbial Composition in Microcosms

**Figure 3:**
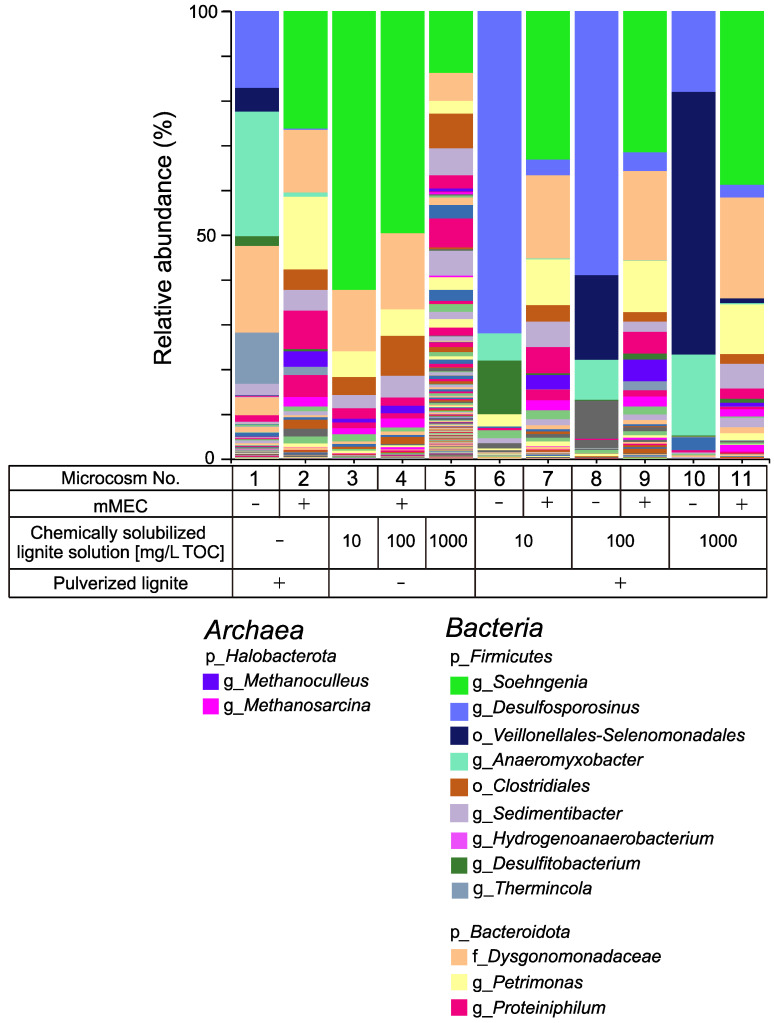
Microbial community structure of each microcosm experiment using mMEC. Major taxonomic groups with a relative abundance of >c.a. 1.0% were indicated. p—Phylum level; o—Order level; f—Family level; g—Genus level. −, not added or absent; +, added or present.

The microbial composition of the microcosms was investigated. Total DNA was extracted from the microcosms on the final incubation day and NGS analysis was performed. The highest value of the Chao 1 index and the archaeal and bacterial ASVs were observed in microcosm No. 5 (Supplementary Table S3), indicating that its archaeal and bacterial richness and diversity are higher than any other microcosm. Two genera *Methanosarcina* (0.03–1.5%) and *Methanoculleus* (0.6–4.8%), were detected as the major archaeal groups in microcosms to which mMEC was added, with the methane production being observed (Figure 3 and Supplementary Table S6). These two major archaeal groups were also detected in the MECs (Figure 1). Only five or fewer ASVs of *Archaea* ASVs were observed in microcosms without the mMEC, whereas ten or more ASVs of *Archaea* were observed in microcosms with the mMEC (Supplementary Table S3).

The following order and genera were detected only in the microcosms amended with pulverized lignite as a CM: *Desulfosporosinus* and *Anaeromyxobacter* in microcosms No. 1, 2, and 8-11; the genus *Thermincola* in microcosms No. 1, 2, 7, 9, and 11; and *Veillonellales-Selenomonadales* in microcosms No. 1, 8, 10, and 11. Bacteria belonging to these orders and genera are indigenous to the lignite used in this study.

### 3.2. Methane Production in Microcosms Supplemented with Chemically Solubilized Lignite Solution Using the Microbial Consortium, SAL25-2

#### 3.2.1. Methane Production Using the SAL25-2

**Figure 4:**
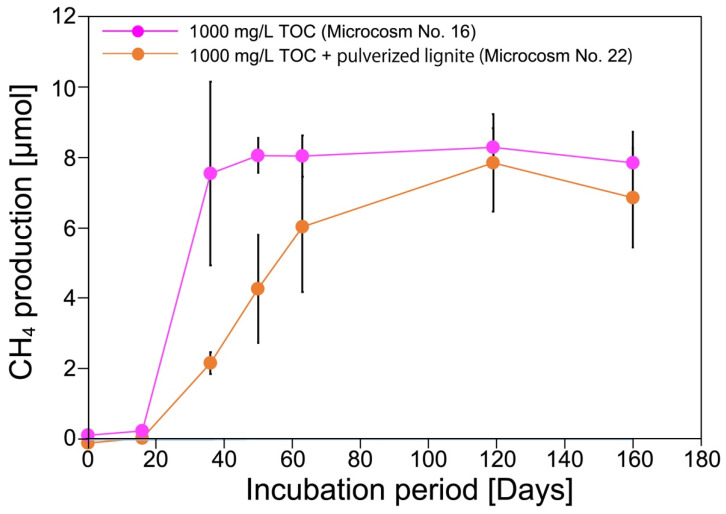
Methane production in microcosms using SAL25-2. Only results of microcosms amended with chemically solubilized lignite solution with 1000 mg/L TOC were shown in this figure (Microcosms No. 16 and 22) because little to no methane was produced during the incubation period of the current study when 100 or 10 mg/L TOC of chemically solubilized lignite solution was used.

Methane production was examined using another type of microbial consortium obtained from in situ groundwater, SAL25-2. As depicted in Figure 4, methane was successfully produced when the chemically solubilized lignite solution with 1000 mg/L TOC was supplemented, with approximately 8 µmol of CH_4_ being produced at maximum (microcosm Nos. 16 and 22), and no significant difference in the maximum methane production was observed between those microcosms. No methane production was observed when the chemically solubilized lignite solution with 100 or 10 mg/L TOC was supplemented (microcosms No. 14, 15, 18, and 20) or when SAL25-2 was absent (microcosms No. 12, 17, 19, and 21).

We examined the enhancement effect of pulverized lignite as a CM on methane production in the microcosms (microcosms No. 17–22). Methane was produced when the chemically solubilized lignite solution with 1000 mg/L TOC was added (microcosm No. 22) (Figure 4). The conversion rate of TOC to methane in microcosms No. 16 and 22 was calculated to be approximately 0.08% when the maximum CH_4_ production (approximately 8 µmol) was attained. No difference in the conversion rate was observed between microcosms No. 16 and 22. Contrary to the results obtained using mMEC, no methane production enhancement was observed (Figure 4). Pulverized lignite addition seemed to inhibit methane production until it reached a plateau level.

#### 3.2.2. Microbial Composition in Microcosms

**Figure 5:**
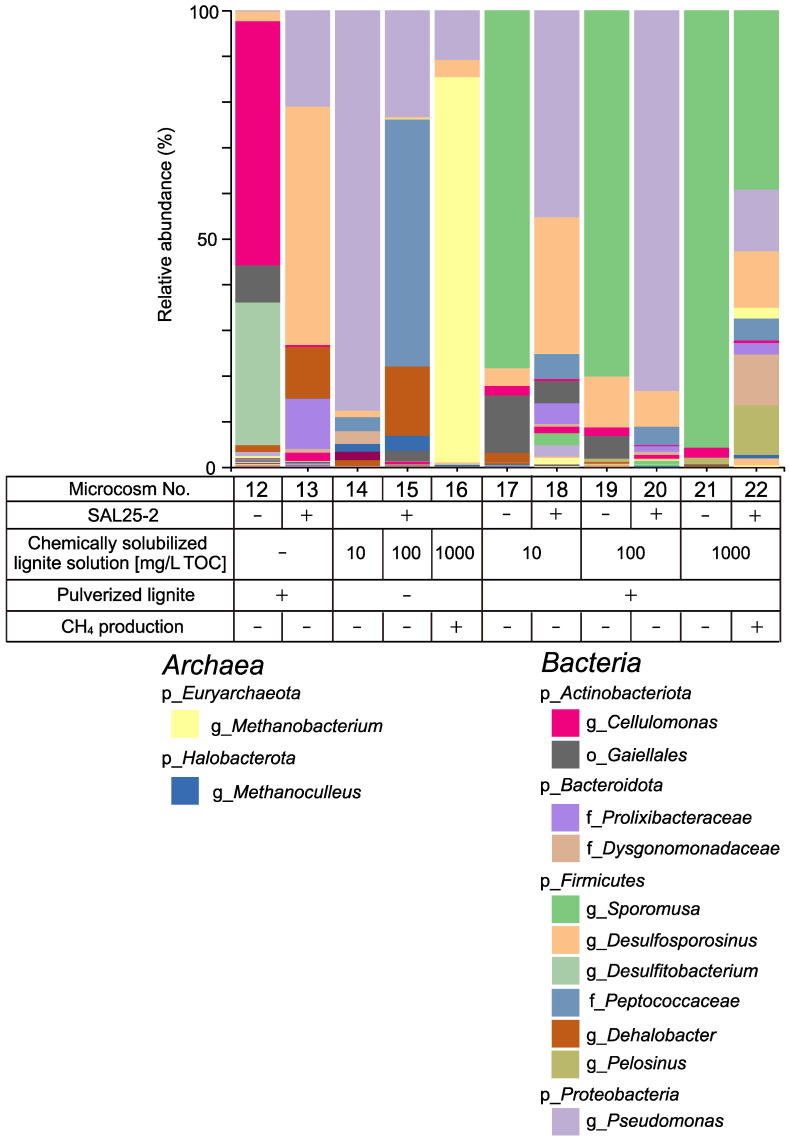
Microbial community structure of each microcosm experiment using SAL25-2. Microcosm numbers correspond to those in Supplementary Tables S4 and S7. Major taxonomic groups with a relative abundance of >c.a. 1.0% were indicated. The methane production results were confirmed in Figure 4. p—Phylum level; o—Order level; f—Family level; g—Genus level. −, not added or absent; +, added or present.

The microbial composition in the microcosms was examined using NGS (Figure 5). The domain *Archaea* was detected in microcosms No. 13, 15, 16, 18, 20, and 22. Regarding the microcosms from which methane production was observed, the relative abundance of the genus *Methanobacterium* was 84.4% in microcosm No. 16, which represented a major part of the microbial community structure, whereas it was only 2.3% in microcosm No. 22 (Supplementary Table S7). The relative abundance of the genus *Methanoculleus* was 3.2 and 0.8% in microcosms No. 15 and No. 22, respectively (Supplementary Table S7). In microcosm No. 16, the genus *Methanoculleus* was below the detection level. The genus *Methanobacterium* in the domain *Archaea* and the genera *Pseudomonas* and *Desulfosporosinus* in the domain *Bacteria* were the main three groups in microcosm No. 16. The genera *Methanobacterium* and *Methanoculleus* in the domain *Archaea* were the main methanogens. The lowest Chao1 index value was observed in microcosm No. 16 (Supplementary Table S4). This indicates that microcosm No. 16 was less diverse than microcosm No. 22.

### 3.3. Real-Time Quantitative PCR Analysis of Microcosms

**Figure 6:**
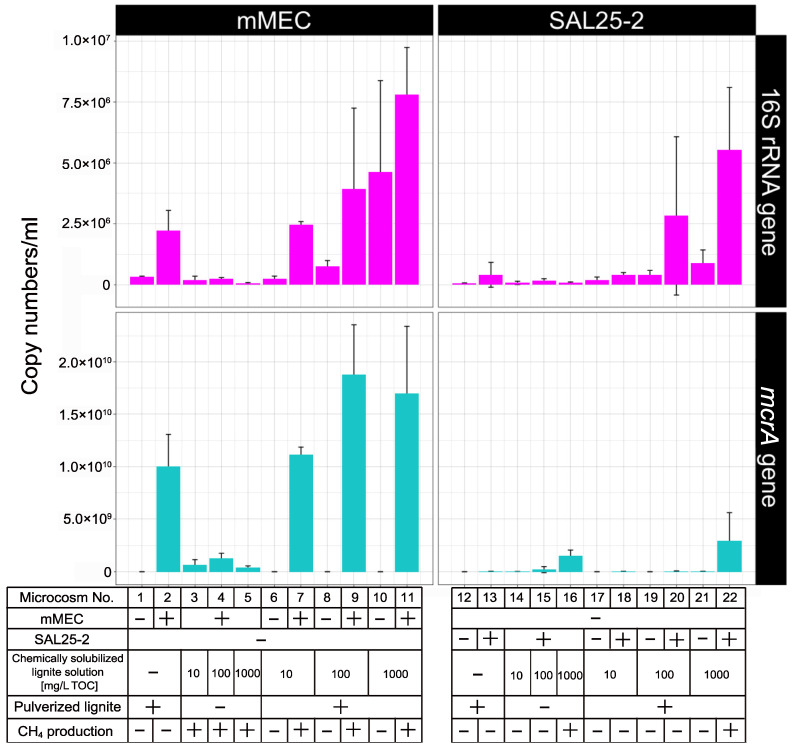
Copy numbers of 16S rRNA and *mcrA* genes in the microcosms using the mMEC (microcosms No. 1–11) and SAL25-2 (microcosms No. 12–22) determined by real-time qPCR. Bars represent mean value ± std. of triplicate samples. −, not added or absent; +, added or present.

Real-time qPCR was conducted to quantify the copy numbers of 16S rRNA gene for the domain *Bacteria* and the methanogenic archaeal *mcrA* gene. Results are shown in Figure 6 and Supplementary Table S8. The copy numbers of 16S rRNA gene in microcosms No. 1 and 12 were 3.22 × 10^5^ ± 0.28 × 10^5^ and 5.80 × 10^4^ ± 1.82 × 10^4^ copies/mL, respectively. The 16S rRNA gene abundance in microcosms No. 1 and 12 would be attributed to the bacteria indigenous to lignite. The *mcrA* gene in those microcosms was under detection level.

When pulverized lignite was added to the microcosms, the copy numbers of 16 S rRNA and *mcrA* genes increased. In the microcosms using mMEC, the increase of the copy numbers of 16 rRNA and *mcrA* genes was approximately 13- and 20-fold, respectively, when microcosms No. 3, 4, and 5 were compared with No. 7, 9, and 11. In the microcosms using SAL25-2, the increase of the copy numbers of 16 rRNA and *mcrA* genes was approximately 28- and 1.8-fold, respectively, when microcosms No. 14, 15, and 16 were compared with No. 18, 20, and 22.

Among the mMEC-inoculated microcosms, the copy numbers of 16S RNA and *mcrA* genes in microcosms No. 5 and 11, to which the chemically solubilized lignite solution with 1000 mg/L TOC was added, were compared. The copy numbers of 16S RNA and *mcrA* genes in microcosm No. 5 were 6.43 × 10^4^ ± 2.60 × 10^4^ and 0.41 × 10^9^ ± 0.15 × 10^9^ copies/mL, respectively, and those in microcosm No. 11 were 7.82 × 10^6^ ± 1.92 × 10^6^ and 1.70 × 10^10^ ± 0.64 × 10^10^, respectively. The increase rates of copy numbers between microcosm No. 5 and No. 11 were approximately 122-fold in 16S RNA gene and 41-fold in *mcrA* gene.

Among the SAL25-2-inoculated microcosms, the copy numbers of 16S RNA and *mcrA* genes in microcosms No. 16 and 22, to which the chemically solubilized lignite solution with 1,000 mg/L TOC was added, were also compared. The copy numbers of 16S RNA and *mcrA* genes in microcosm No. 16 were 7.97 × 10^4^ ± 3.35 × 10^4^ and 1.50 × 10^9^ ± 0.56 × 10^9^ copies/mL, respectively, and those in microcosm No. 22 were 5.53 × 10^6^ ± 2.56 × 10^6^ and 2.95 × 10^9^ ± 2.65 × 10^9^ copies/mL, respectively. The increase rates of copy numbers between microcosm No. 16 and No. 22 were approximately 69-fold in 16S RNA gene and 2-fold in *mcrA* gene.

## 4. Discussion

In the concept of the Subsurface Cultivation and Gasification (SCG) method, there is an additional injection of reagent/minerals/microorganisms following the injection of H_2_O_2_ solution [46,49,61]. This concept was conceived to utilize unused subterranean coal in situ to produce methane. As other studies have also shown, the rate-limiting step is to convert the recalcitrant organic matter in coal to lower-molecular-weight organic compounds that are readily bioavailable [11,43]. We examined bioaugmentation and the effect of pulverized lignite as a solid phase on methanogenesis acceleration using a chemically solubilized lignite solution as a carbon source. Bioaugmentation (microbial augmentation) is the process of adding microorganisms to the coal seams to enhance or promptly initiate microbial CBM production [10]. This is one of the most promising strategies for converting organic matter into methane [62]. In this study, we used two types of microbial consortia: mMEC and SAL25-2, along with pulverized lignite as a CM. As shown in Figure 2 and Figure 4, adding mMEC or SAL25-2 to the microcosms produced methane from the chemically solubilized lignite solution. Those results demonstrated that bioaugmentation using microbial consortia is an effective way to produce methane from chemically solubilized lignite solution.

Solubilization of coal by chemical agents is an effective pretreatment to produce low molecular weight substances which are then utilized for microbial methane production. Several chemical agents, such as surfactants [13,18], oxidants [44], acids [43], bases [43], and chelating agents, have been used. In addition to those chemicals, hydrogen peroxide (H_2_O_2_) is one of the most effective solubilizing agents for coal [37,38,63,64]. The H_2_O_2_ oxidation of lignite produces malonic and succinic acids [63], as well as acetic and formic acids [50]. Since the latter two acids can be utilized as substrates for methanogens [64], H_2_O_2_ is one of the best solubilizing agents for lignite to produce low molecular weight organic molecules. Furthermore, it is very important to minimize the environmental pollution arising from this process, considering the application of H_2_O_2_ to the SCG method. The decomposition of H_2_O_2_ produces water and oxygen, which are non-polluting agents, according to the following reaction (2H_2_O_2_ → 2H_2_O + O_2_). As H_2_O_2_ is generally considered a non-polluting and clean oxidant [45], we utilized H_2_O_2_ to produce chemically solubilized lignite solution for the future application of H_2_O_2_ in the SCG method.

The TOC concentration of the chemically solubilized lignite solution was important for the success of methane production using microbial consortia. We examined different concentrations of chemically solubilized lignite solutions, namely 1000, 100, and 10 mg/L TOC. Methane production was observed in the microcosms amended with 1,000 mg/L TOC of the chemically solubilized lignite solution. However, little to no methane production was observed during the incubation period of the current study when 10 mg/L TOC of chemically solubilized lignite solution was used. This means that the higher the concentration of the chemically solubilized lignite solution, the more methane is produced. When lignite reacts with H_2_O_2_, organic substances, such as acetate, formate, and other organic acids (succinate and malonate), are produced [50]. Acetate and formate are potential substrates for methanogens [65,66]. Increasing the TOC concentration of the chemically solubilized lignite solution would lead to successful methanogenesis. Xiao et al., (2013) reported that a higher acetate concentration (over 1619.47 mg acetate L^−1^) inhibited the utilization rate of acetate by methanogens in the methanogenic phase of a two-phase anaerobic process [67]. In their study, no inhibitory effect of acetate was observed between 546.08 and 1619.47 mg acetate L^−1^. In this study, no obvious inhibitory effect of acetate on methanogenesis was observed. The initial acetate concentration in the microcosms was calculated to be 334.8 mg/L when a chemically solubilized lignite solution with 1000 mg/L TOC was added (Table 1).

One of the interesting points of this study was that adding pulverized lignite significantly enhanced methane production when mMEC was used (Figure 2). The typical methane production reactions from major methanogenic substrates have been reported [65,68]. We thought that methane would be mainly produced from acetate and formate in chemically solubilized lignite solution, although it is still unclear if all the acetate and formate would be utilized as a substrate for methanogenesis. Based on the calculations in Table 1, 55.8 µmol of acetate and 50.9 µmol of formate were contained in microcosms No. 5 and 11. If all methane was produced from acetate and formate in the chemically solubilized lignite solution, theoretically, 68.5 µmol of methane would be produced. In microcosm No. 11, the highest methane production was 115.9 ± 22.9 µmol on day 56 (Figure 2), which was higher than the theoretically produced methane from acetate and formate. These results indicated that methane might be produced from other organic substances, along with acetate and formate. The conversion rates to methane from TOC in microcosms No. 7, 9 and 11 (0–13.8%) were higher than those in microcosms No. 3, 4, and 5 (0–2.8%) (Supplementary Table S2c). Adding pulverized lignite might facilitate the utilization of other organic substances in chemically solubilized lignite solutions, along with acetate and formate. Compared with the microcosms without pulverized lignite, the methane production in the microcosms with pulverized lignite was enhanced approximately 10-fold between microcosms No. 9 and 11 (Figure 2). When microcosm No. 5 was regarded as the control experiment for microcosm No. 11 in terms of the effect of the addition of pulverized lignite as a CM on methane production, methane production was enhanced by approximately 50-fold. To the best of our knowledge, the 50-fold increase was the highest number of times that methane production increased relative to the control, compared to the data in the review article that Martins et al., (2018) summarized as the enhancement or inhibition of methane production by CM use [69]. Salvador et al., (2017) reported that the initial methane production rate of *Methanobacterium formicicum* cultures increased up to 17 times, with 5 g/L carbon nanotubes (CNT) [70]. Dang et al., (2017) reported that the lag period for methane production was significantly reduced in reactors with granular activated carbon (GAC) and the cumulative methane production was 18 times higher than that of the control [71]. Konieczna et al., (2021) studied the relationship between energy efficiency and greenhouse gas (GHG) emissions to the atmosphere from the silage maize plantation technologies [72]. They reported that the higher the energy efficiency of silage maize plantations, the lower the air pollution emissions in the form of GHG. Our main focus is to improve methane production. Adding pulverized lignite could improve the energy efficiency of producing methane.

Contrary to the microcosm experiment using mMEC, methane production was not enhanced when SAL25-2 was used (Figure 5). However, adding pulverized lignite increased the copy numbers of both 16S rRNA and *mcrA* (microcosms No. 16 and 22 in Figure 6). One plausible explanation is that bacteria might outcompete archaea for growth substrates although the copy number of *mcrA* gene was slightly higher in microcosm No. 22 (pulverized lignite-added microcosm) than that in microcosm No. 16. Previous studies have suggested that adding CM, such as magnetite, promotes the direct interspecies electron transfer (DIET), leading to syntrophic metabolism improvement [69,73,74] and the enhancement of methanogenesis under harsh environmental conditions, such as high ammonia and hydrogen sulfide concentrations [75,76]. It has been reported that the most commonly studied DIET-implicated genera are *Geobacter* and *Shewanella* [73,77,78]. The genera *Geobacter* and *Shewanella* were below the detection level in mMEC (Figure 1 and Figure 3; Supplementary Table S6) and a minor fraction in SAL25-2 (Supplementary Table S7). Therefore, it is less likely that DIET is the main reason for the increase in methane production and copy numbers of 16S rRNA and *mcrA* using mMEC and SAL25-2. Guo et al., (2018) examined the adsorption rate of methanogens on the surface of different coal samples using spectrophotometry. They reported that an increase in methane formation was associated with higher cell adsorption of methanogens on coal surfaces [79]. In their study, a molecular biological approach was not utilized to quantify the number of microorganisms. Hazrin-Chong et al., (2021) examined the influence of surface physicochemical properties on microbial cell attachment to different coal types with varying surface properties, utilizing a known coal-oxidizing bacterium, *Pseudomonas fluorescens* PF-5. They concluded that the lignite coal surface had the highest number of cells attached [80]. In our microcosm study, the solid-to-liquid ratio was 1 to 10 (1/10). The surface of pulverized lignite would be an adequate location for the growth of archaea and bacteria.

According to the real-time qPCR analysis (Figure 6), the copy numbers of both the 16S rRNA and *mcrA* genes were higher in the microcosms supplemented with pulverized lignite (microcosms No. 7, 9, and 11) than in the microcosms without pulverized lignite (microcosms No. 3, 4, and 5). Among the mMEC-inoculated microcosms, the increase rates of the copy numbers of 16 rRNA and *mcrA* genes were approximately 13- and 20-fold, respectively, when microcosms No. 3, 4, and 5 were compared with No. 7, 9, and 11. Among the SAL25-2 inoculated microcosms, similar results were also observed. The increase rates of the copy numbers of 16 rRNA and *mcrA* genes were 28- and 1.8-fold, respectively, when microcosms No. 14, 15, and 16 were compared with No. 18, 20, and 22. One of the possible functions of pulverized lignite is as a site for the growth of microorganisms. Lignite consists of organic macromolecules, which are generally recalcitrant to biodegradation in nature. Once a part of lignite is degraded by microbial activities such as fermentation and hydrolysis, low molecular weight organic molecules would be produced. Those molecules, in turn, would be bioavailable for growth [16,28]. Inagaki et al., (2015) reported that they detected a higher cellular concentration range in coal-bearing horizons than in any other sediment [81]. Many microorganisms form biofilms on a wide variety of solid surfaces, including coal [80,82]. It has been reported that the number of bacteria on surfaces was drastically higher than in the surrounding liquid environment [83]. Furthermore, our qPCR analysis showed that the copy numbers of 16S rRNA and *mcrA* genes were higher in microcosms with pulverized lignite than those without it (Figure 6). These results indicate that pulverized lignite as a CM would play a role as a solid surface for biofilm formation and facilitate the growth of archaea and bacteria in the microcosms, leading to an increase in methane production.

Another possible explanation for the increase in methane using mMEC might be the difference in methanogens. The mixture of methanogenic consortia, mMEC, comprises five MECs (No. 35, 36, 37, 45, and 46), which have been maintained in our laboratory for several years (Figure 1). The major methanogens in the mMEC were the genera *Methanosarcina* and *Methanoculleus*, whereas they were the genus *Methanobacterium* in SAL25-2 (Figure 3 and Figure 5). The genus *Methanosarcina* was below the detection level for SAL25-2 (Figure 5). *Methanosarcina* uses various substances, including H_2_/CO_2_, methanol, methylamines, and acetate [84]. *Methanoculleus* and *Methanobacterium* are hydrogenotrophic. Since the chemically solubilized lignite solution contains a low molecular weight of organic substances, such as acetate and formate, the solution might be a more favorable substrate for the methanogens in mMEC than those in SAL25-2, resulting in a higher methane production in the microcosms inoculated with mMEC. *Methanosarcina* species possess a unique membrane-bound electron carrier, methanophenazine (Mph), which plays the same role as quinones in the electron transport chain [85]. Fu et al., examined the effect of magnetite nanoparticles (nanoFe_3_O_4_) as CM on the enhancement of methane production using three pure cultures: two hydrogenotrophic methanogens (*ethanococcus maripaludis* and *Methanocella conradii*), and one acetotrophic methanogen (*Methanosarcina barkeri*) [86,87]. In their study, nano-Fe_3_O_4_ addition did not affect methane production by *Methanococcus maripaludis* and *Methanocella conradii*, whereas nanoFe_3_O_4_ significantly promoted methane production by *Methanosarcina barkeri*. Although they did not have a clear discussion, they described that the most essential difference between the former two hydrogenotrophic methanogens and the latter acetotrophic methanogens was the lack of an electron transport chain in the membrane [87]. Several other studies have suggested that *Methanosarcina* species can accept electrons from nonbiological extracellular surfaces [88,89]. In our study, the genus *Methansarcina* was one of the main methanogens in mMEC (Figure 1 and Figure 3, Supplementary Table S6). Adding pulverized lignite might enhance methane production more pronouncedly in mMEC-amended microcosms than in SAL25-2-amended ones by promoting electron transport. Microbial community composition varies depending on the natural settings. The composition of the microbial community structure in the in situ environment is an important factor in the success of methanogenesis in the subsurface environment.

## 5. Conclusions

We believe that chemically solubilized lignite treated with H_2_O_2_ is one of the most favorable ways to effectively provide growth substrates for methanogens. The organic matter released from the lignite and the remaining H_2_O_2_ after the reaction with lignite had no detrimental effect on methanogen growth and methane production. The chemical solubilization of lignite is conducive to the acceleration of methane production from recalcitrant organic matter in coal. Additionally, bioaugmentation using a methanogenic consortium, which is synonymous with microbially enhanced coalbed methane (MECBM) production, could be an alternative to facilitate methane production from chemically solubilized lignite. The addition of pulverized lignite as CM could greatly enhance methane production with the increase in conversion rate of TOC to methane, depending on the composition of the microbial community structures. Methane production using mMEC was drastically enhanced by approximately 50–folds, compared to that of the control. To the best of our knowledge, this is the highest number of times methane production increased relative to the control.

## Figures and Tables

**Table 1:** Characteristics of chemically solubilized lignite solution.

Parameter	Original Solution	Concentrations in Microcosms		
pH	2.4			
Dissolved total organic carbon [mg/L TOC]	2800	1000	100	10
Dissolved nitrogen [mg/L]	145	52.0	5.2	0.5
Acetate [mg/L]	937	335	33.5	3.3
Formate [mg/L]	656	235	23.5	2.3
Cl^−^ [mg/L]	5.40	1.9	0.2	0.02
NO_3_^−^ [mg/L]	7.50	2.7	0.3	0.03
SO_4_^2−^ [mg/L]	102	36.4	3.6	0.4
Malonate [mg/L]	1060	378	37.8	3.8
Succinate [mg/L]	241	86.0	8.6	0.9
Oxalate [mg/L]	1010			
H_2_O_2_ [%]	<10^−4^			

## Data Availability

The datasets analyzed during the present study are available at the DNA Data Bank of Japan (DDBJ). BioProject Accession Number PRJDB12850. The Sequence Read Archive (DRA) accession numbers are DRA013976, DRA013977, and DRA013978.

## Supplementary Materials

The following supporting information can be downloaded at: https://www.mdpi.com/article/10.3390/microorganisms10101984/s1, Figure S1: Enrichment cultivation of each MEC; Figure S2: Preparation of the mMEC from 5 types of MECs; Table S1: Alpha diversity analysis of MECs; Table S2: Methane production in the microcosms using mMEC; Table S3: Alpha diversity analysis of each microcosm amended with the mMEC; Table S4: Alpha diversity analysis of each microcosm amended with SAL25-2; Table S5: Taxonomy table with the ASV counts and relative abundance in the MECs; Table S6: Taxonomy table with the ASV counts and relative abundance in the microcosms amended with mMEC; Table S7: Taxonomy table with the ASV counts and relative abundance in the microcosms amended with SAL25-2; Table S8: Real-Time quantitative PCR analysis in microcosms.
